# Supplementary material for: Spatiotemporal changes in heat stress exposure in India, 1981-2023
Source: Nat Commun. 2025 Oct 28;16:9496. doi: 10.1038/s41467-025-64840-x (PMC12569015; doi:10.1038/s41467-025-64840-x)
Supplement: Supplementary file 1 — Supplementary Information [file 41467_2025_64840_MOESM1_ESM.docx]

**Supplementary Information**

**Spatiotemporal changes in heat stress exposure in India, 1981-2023**


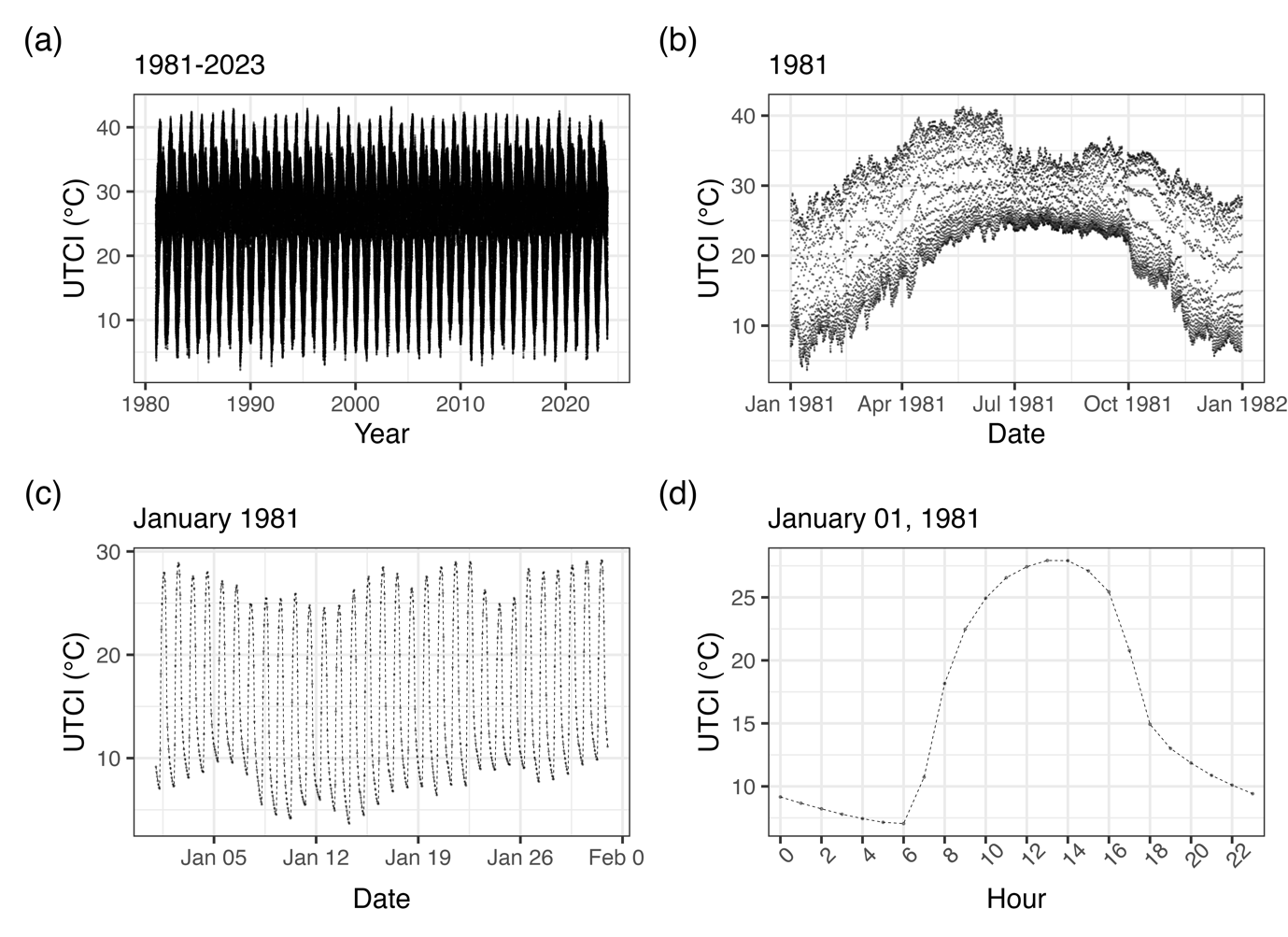


Supplementary Figure 1: The figure plots the hourly UTCI time series for India (averaged across all pixels in the country) for different time scales. (a) January 1, 1981 to December 31, 2023. (b) January 1, 1981 to December 31, 1981. (c) January 1, 1981 to January 31, 1981. (d) January 1, 12:00 am to January 1, 11:00 pm

Supplementary Table 1: Mapping of states and districts to regions in India

| **Region** | **States** | **Districts** |
| --- | --- | --- |
| Central | Chhattisgarh, Madhya Pradesh | Balrampur - Chhattisgarh, Koriya, Jashpur, Raigarh, Korba, Janjgir-Champa, Kabeerdham, Rajnandgaon, Mahasamund, Dhamtari, Uttar Bastar Kanker, Narayanpur, Bijapur - Chhattisgarh, Sheopur, Morena, Bhind, Gwalior, Datia, Shivpuri, Tikamgarh, Chhatarpur, Panna, Sagar, Damoh, Satna, Rewa, Umaria, Neemuch, Mandsaur, Ratlam, Ujjain, Dewas, Dhar, Indore, Khargone (West Nimar), Barwani, Rajgarh, Vidisha, Bhopal, Sehore, Raisen, Betul, Harda, Hoshangabad, Katni, Jabalpur, Narsimhapur, Dindori, Mandla, Chhindwara, Seoni, Balaghat, Guna, Ashoknagar, Shahdol, Anuppur, Singrauli, Jhabua, Alirajpur, Khandwa (East Nimar), Burhanpur, Balod, Baloda Bazar, Bastar, Bemetara, Bilaspur, Dantewada, Durg, Gariyaband, Kodagaon, Mungeli, Raipur, Sukma, Surguja, Agar Malwa, Shajapur, Surajpur, Sidhi |
| East | West Bengal, Bihar, Sikkim, Arunachal Pradesh, Nagaland, Manipur, Mizoram, Tripura, Meghalaya, Assam, Jharkhand, Odisha | Kolkata, Pashchim Champaran, Purba Champaran, Sheohar, Sitamarhi, Madhubani, Supaul, Araria, Kishanganj, Purnia, Katihar, Madhepura, Saharsa, Darbhanga, Muzaffarpur, Gopalganj, Siwan, Saran, Vaishali, Samastipur, Begusarai, Khagaria, Bhagalpur, Banka, Munger, Lakhisarai, Sheikhpura, Nalanda, Patna, Bhojpur, Buxer, Kaimur (Bhabua), Rohtas, Gaya, Nawada, Jamui, Jehanabad, Arwal, North District, West District, South District, East District, Tawang, West Kameng, East Kameng, Papum Pare, Upper Subansiri, Upper Siang, Changlang, Lower Subansiri, Dibang Valley, Lower Dibang Valley, Anjaw, Mon, Mokokchung, Zunheboto, Wokha, Dimapur, Phek, Tuensang, Longleng, Kiphire, Kohima, Peren, Senapati, Tamenglong, Churachandpur, Bishnupur, Thoubal, Imphal West, Imphal East, Ukhrul, Chandel, Mamit, Kolasib, Aizawl, Champhai, Serchhip, Lunglei, Lawngtlai, Dhalai, South Garo Hills, Ribhoi, East Khasi Hills, Kokrajhar, Goalpara, Barpeta, Morigaon, Lakhimpur, Dhemaji, Tinsukia, Dibrugarh, Golaghat, Dima Hasao, Cachar, Karimganj, Hailakandi, Bongaigaon, Chirang, Kamrup, Kamrup Metropolitan, Nalbari, Baksa, Darrang, Udalguri, Darjiling, Jalpaiguri, Koch Bihar, Uttar Dinajpur, Dakshin Dinajpur, Maldah, Murshidabad, Birbhum, Nadia, North Twenty Four Parganas, Hugli, Bankura, Puruliya, South Twenty Four Parganas, Paschim Medinipur, Purba Medinipur, Garhwa, Chatra, Kodarma, Giridih, Deoghar, Godda, Sahibganj, Pakur, Dhanbad, Bokaro, Lohardaga, Purbi Singhbhum, Palamu, Latehar, Hazaribagh, Ramgarh, Dumka, Jamtara, Ranchi, Khunti, Gumla, Simdega, Pashchimi Singhbhum, Saraikela-Kharsawan, Bargarh, Jharsuguda, Sambalpur, Debagarh, Sundargarh, Kendujhar, Mayurbhanj, Baleshwar, Bhadrak, Kendrapara, Cuttack, Jajapur, Dhenkanal, Anugul, Nayagarh, Khordha, Puri, Ganjam, Gajapati, Kandhamal, Baudh, Subarnapur, Balangir, Nuapada, Kalahandi, Rayagada, Nabarangapur, Koraput, Malkangiri, East Siang, Kra Daadi, Kurung Kumey, Lohit, Longding, Namsai, Siang, Tirap, West Siang, Biswanath, Charaideo, Dhubri, Hojai, Jorhat, Karbi Anglong, Majuli, Nagaon, Sivasagar, Sonitpur, South Salmara Mancachar, West Karbi Anglong, East Garo Hills, East Jantia Hills, North Garo Hills, South West Garo Hills, South West Khasi Hills, West Garo Hills, West Jaintia Hills, West Khasi Hills, Gomati, Khowai, North Tripura, South Tripura, Unakoti, West Tripura, Paschim Barddhaman, Purba Barddhaman, Sepahijala, Saiha, Haora, Jagatsinghapur, Aurangabad - Bihar |
| North | Jammu & Kashmir, Ladakh, Himachal Pradesh, Punjab, Chandigarh, Uttarakhand, Haryana, Uttar Pradesh, NCT Of Delhi | Muzaffarabad, Kupwara, Badgam, Leh, Punch, Rajouri, Kathua, Baramula, Bandipore, Srinagar, Ganderbal, Pulwama, Shupiyan, Anantnag, Kulgam, Doda, Ramban, Kishtwar, Udhampur, Reasi, Jammu, Samba, Chamba, Kangra, Lahul & Spiti, Kullu, Mandi, Hamirpur, Una, Bilaspur, Solan, Sirmaur, Shimla, Kinnaur, Kapurthala, Jalandhar, Hoshiarpur, Shahid Bhagat Singh Nagar, Fatehgarh Sahib, Ludhiana, Moga, Muktsar, Faridkot, Bathinda, Mansa, Patiala, Amritsar, Tarn Taran, Rupnagar, Sahibzada Ajit Singh Nagar, Sangrur, Barnala, Chandigarh, Uttarkashi, Chamoli, Rudraprayag, Tehri Garhwal, Dehradun, Garhwal, Pithoragarh, Bageshwar, Almora, Champawat, Nainital, Udham Singh Nagar, Hardwar, Panchkula, Ambala, Yamunanagar, Kurukshetra, Kaithal, Karnal, Panipat, Sonipat, Jind, Fatehabad, Sirsa, Hisar, Rohtak, Jhajjar, Mahendragarh, Rewari, Gurgaon, Mewat, Faridabad, Palwal, Saharanpur, Bijnor, Rampur, Jyotiba Phule Nagar, Meerut, Baghpat, Gautam Buddha Nagar, Bulandshahr, Aligarh, Mahamaya Nagar, Mathura, Agra, Firozabad, Mainpuri, Bareilly, Pilibhit, Shahjahanpur, Sitapur, Hardoi, Unnao, Lucknow, Farrukhabad, Kannauj, Etawah, Auraiya, Kanpur Dehat, Kanpur Nagar, Jalaun, Jhansi, Lalitpur, Hamirpur, Mahoba, Banda, Chitrakoot, Fatehpur, Pratapgarh, Kaushambi, Allahabad, Bara Banki, Faizabad, Ambedkar Nagar, Bahraich, Shrawasti, Gonda, Siddharthnagar, Basti, Sant Kabir Nagar, Maharajganj, Gorakhpur, Kushinagar, Deoria, Azamgarh, Mau, Ballia, Jaunpur, Ghazipur, Chandauli, Varanasi, Sant Ravidas Nagar, Mirzapur, Sonbhadra, Etah, Kanshiram Nagar, Balrampur - Uttar Pradesh, Central, East, New Delhi, North, North East, North West, Shahdara, South, South East, South West, West, Bhiwani, Charkhi Dadri, Fazilka, Firozpur, Gurdaspur, Pathankot, Amethi, Budaun, Ghaziabad, Hapur, Moradabad, Muzaffarnagar, Rae Bareli, Sambhal, Shamli, Sultanpur, Kheri, Kargil, Mirpur |
| South | Andhra Pradesh, Karnataka, Kerala, Tamil Nadu, Puducherry, Telangana, Lakshadweep, Andaman & Nicobar Islands | Alluri Sitharama Raju, Anakapalli, Ananthapuramu, Annamayya, Bapatla, Chittoor, Dr. B.r. Ambedkar Konaseema, East Godavari, Eluru, Guntur, Kakinada, Krishna, Kurnool, Kurnool, Ntr, Palnadu, Parvathipuram Manyam, Prakasam, Sri Potti Sriramulu Nellore, Sri Sathya Sai, Srikakulam, Tirupati, Visakhapatnam, Vizianagaram, West Godavari, Y.s.r., Belgaum, Bagalkot, Bijapur - Karnataka, Bidar, Raichur, Koppal, Gadag, Dharwad, Uttara Kannada, Haveri, Bellary, Chitradurga, Davanagere, Shimoga, Chikmagalur, Tumkur, Bangalore, Mandya, Hassan, Dakshina Kannada, Kodagu, Mysore, Chamarajanagar, Gulbarga, Yadgir, Kolar, Chikkaballapura, Bangalore Rural, Ramanagara, Wayanad, Kozhikode, Malappuram, Palakkad, Thrissur, Ernakulam, Idukki, Kottayam, Pathanamthitta, Kollam, Thiruvananthapuram, Chennai, Kancheepuram, Vellore, Tiruvannamalai, Viluppuram, Salem, Namakkal, Erode, The Nilgiris, Dindigul, Karur, Tiruchirappalli, Perambalur, Ariyalur, Cuddalore, Nagapattinam, Thiruvarur, Thanjavur, Pudukkottai, Sivaganga, Madurai, Theni, Virudhunagar, Thoothukkudi, Tirunelveli, Kanniyakumari, Dharmapuri, Krishnagiri, Coimbatore, Tiruppur, Yanam, Puducherry, Adilabad, Bhadradri Kothagudem, Hyderabad, Jagitial, Jangoan, Jayashankar Bhupalapally, Jogulamba Gadwal, Kamareddy, Karimnagar, Khammam, Komaram Bheem Asifabad, Mahabubabad, Mahabubnagar, Mancherial, Medak, Medchal-Malkajgiri, Nagarkurnool, Nalgonda, Nirmal, Nizamabad, Peddapalli, Rajanna Sircilla, Ranga Reddy, Sangareddy, Siddipet, Suryapet, Vikarabad, Wanaparthy, Warangal Rural, Warangal Urban, Yadadri Bhuvanagiri, Thiruvallur, Udupi, Kasaragod, Lakshadweep, Kannur, Alappuzha, South Andaman, Ramanathapuram, Nicobars, Karaikal, North & Middle Andaman, Mahe |
| West | Rajasthan, Gujarat, Dadra & Nagar Haveli & Daman & Diu, Maharashtra, Goa | Ganganagar, Hanumangarh, Bikaner, Churu, Jhunjhunun, Alwar, Bharatpur, Dhaulpur, Karauli, Sawai Madhopur, Dausa, Jaipur, Sikar, Nagaur, Jodhpur, Jaisalmer, Barmer, Jalor, Sirohi, Pali, Ajmer, Tonk, Bundi, Bhilwara, Rajsamand, Dungarpur, Banswara, Chittaurgarh, Kota, Baran, Jhalawar, Udaipur, Pratapgarh, Kachchh, Banas Kantha, Patan, Mahesana, Gandhinagar, Porbandar, Anand, Dohad, Narmada, Bharuch, The Dangs, Navsari, Valsad, Tapi, Daman, Dadra & Nagar Haveli, Nandurbar, Dhule, Jalgaon, Buldana, Akola, Washim, Amravati, Wardha, Nagpur, Bhandara, Gondiya, Gadchiroli, Chandrapur, Yavatmal, Nanded, Hingoli, Parbhani, Jalna, Nashik, Mumbai Suburban, Mumbai, Raigarh, Pune, Ahmadnagar, Bid, Latur, Osmanabad, Solapur, Satara, Ratnagiri, Sindhudurg, Kolhapur, Sangli, North Goa, South Goa, Ahmadabad, Aravali, Bhavnagar, Botad, Chhota Udaipur, Devbhumi Dwarka, Gir Somnath, Jamnagar, Junagadh, Kheda, Mahisagar, Morbi, Panch Mahals, Rajkot, Sabar Kantha, Surendranagar, Vadodara, Palghar, Thane, Surat, Amreli, Aurangabad - Maharashtra, Diu |

Supplementary Table 2: Variables used to compute the UTCI^1^

| **Source dataset and variable** | **Description** | **Units** | **Resolution** |
| --- | --- | --- | --- |
| ERA5-Land, Surface solar radiation downwards | Amount of solar (shortwave) radiation reaching the surface of the Earth | J m^−2^ | 0.1° |
| ERA5-Land, Surface net solar radiation | Amount of solar radiation reaching the surface of the Earth (both direct and diffuse) minus the amount reflected by the Earth’s surface | J m^−2^ | 0.1° |
| ERA5, Total sky direct solar radiation at surface | Amount of direct solar radiation reaching the surface of the Earth | J m^−2^ | 0.25° |
| ERA5-Land, Surface thermal radiation do`wnwards | Amount of thermal (longwave) radiation emitted by the atmosphere and clouds that reach the Earth’s surface | J m^−2^ | 0.1° |
| ERA5-Land, Surface net thermal radiation | Amount of net thermal radiation at the surface | J m^−2^ | 0.1° |
| ERA5-Land, 2 m temperature | Temperature of air at 2 m above the surface of the land | K | 0.1° |
| ERA5-Land, 2 m dewpoint temperature | Dewpoint temperature to which the air, at 2 m above the surface of land, would have to be cooled for saturation to occur | K | 0.1° |
| ERA5-Land, 10m u-component of wind | Eastward component of the 10 m wind | m s^−1^ | 0.1° |
| ERA5-Land, 10m v-component of wind | Northward component of the 10 m wind | m s^−1^ | 0.1° |


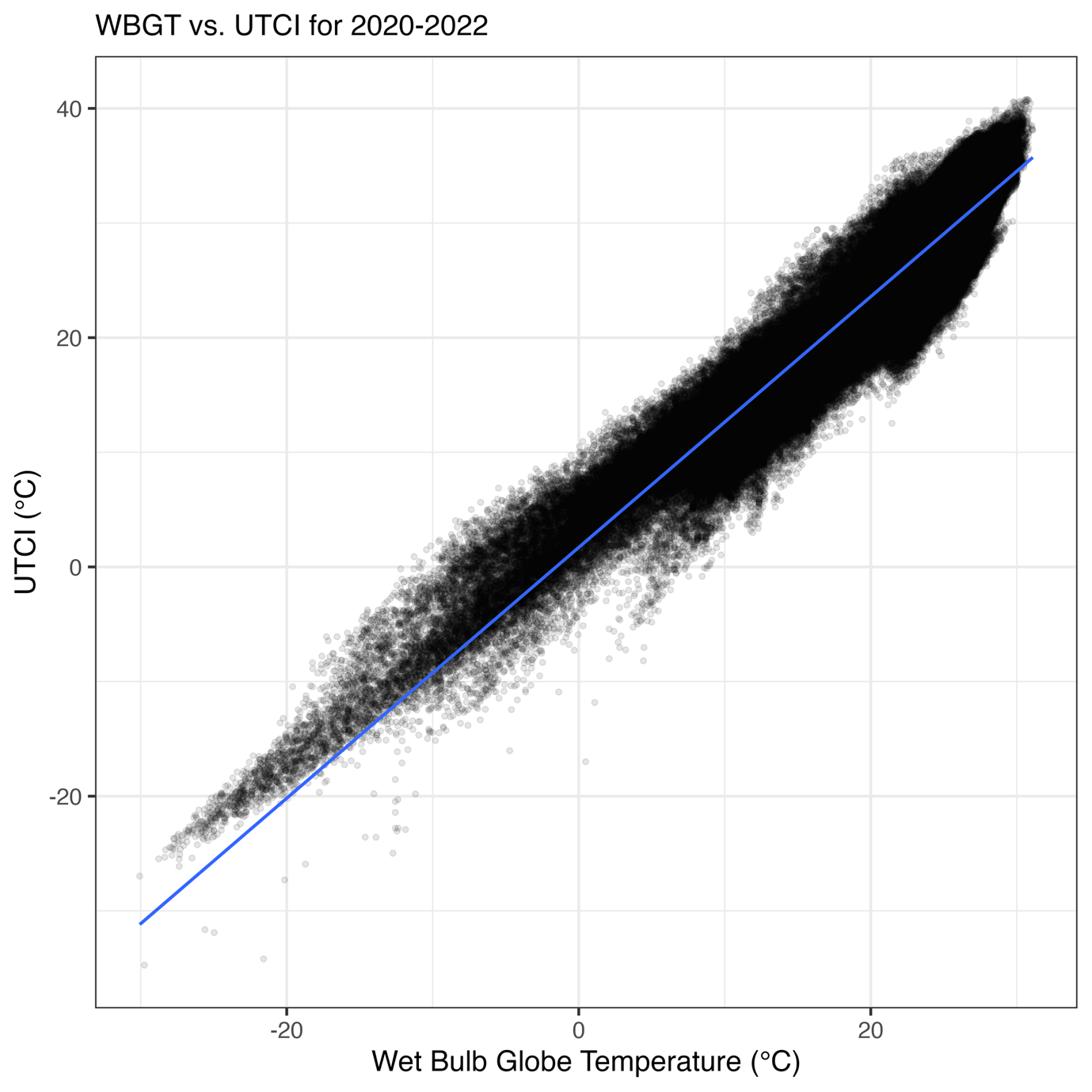


Supplementary Figure 2: The figure plots the correlation between daily average Wet Bulb Global Temperature (WBGT) values at the district level and UTCI values at the district level for 2020-2022. WBGT values are obtained from publicly available data^2^, that is then extracted to the district level. Each point represents a day in a district. The line represent the best fit OLS line for the set of points.

**References**

1. Yan, Y., Xu, Y. & Yue, S. A high-spatial-resolution dataset of human thermal stress indices over South and East Asia. *Scientific data* **8**, 229 (2021).

2. Li, D., Yuan, J. & Kopp, R. E. Escalating global exposure to compound heat-humidity extremes with warming. *Environ. Res. Lett.* **15**, 064003 (2020).
